# Sleep Duration and Screen Time in Young Children with Mild Language Delays

**DOI:** 10.3390/children12111467

**Published:** 2025-10-30

**Authors:** Subul Malik, Melissa Gonzalez, Paris Rayneri, Ruixuan Ma, Ruby Natale, Elana Mansoor

**Affiliations:** 1 Department of Pediatrics, University of Miami Miller School of Medicine, Miami, FL 33136, USA; subul.malik@med.miami.edu (S.M.); mxg1850@med.miami.edu (M.G.); parisrayneri@miami.edu (P.R.); rnatale@med.miami.edu (R.N.); 2 Division of Biostatistics and Bioinformatics, Department of Public Health Sciences, University of Miami Miller School of Medicine, Miami, FL 33136, USA; rxm1424@med.miami.edu

**Keywords:** sleep, screen time, language delays, sociodemographic, preschool-aged children

## Abstract

**Highlights:**

What are the main findings?

- Excessive screen time was significantly associated with lower expressive language scores, while sleep duration showed no direct correlation with language outcomes.
- Sociodemographic factors such as race, insurance status, and caregiver education were strongly correlated with both sleep and screen time behaviors.

What are the implications of the main findings?

- Interventions should address reducing screen time in young children with mild language delays, as findings suggest this modifiable risk factor can impact expressive language development.
- Programs should also consider underlying sociodemographic factors, tailoring interventions to each family to increase adherence to national health guidelines and optimize developmental outcomes.

**Abstract:**

**Background/Objectives**: Excessive screen time and inadequate sleep are well-established developmental risk factors, yet limited research has examined the relationship between adherence to national sleep and screen time guidelines and language outcomes in children with mild language delays. This study examined sleep, screen time, and language outcomes in 765 children aged 1–5 with mild language delays enrolled in the Early Discovery Program. **Methods**: Sleep and screen time were categorized according to American Academy of Pediatrics and Caring for Our Children guidelines. Language outcomes were measured using standardized scores from the Preschool Language Scales–Fifth Edition. **Results**: Sociodemographic factors, including race, insurance status, and caregiver education, were significantly associated with sleep and screen behaviors. Excessive screen time was negatively associated with expressive language scores, while sleep duration showed no significant relationship. **Conclusions**: Findings highlight the need for early interventions that promote adherence to national health guidelines and address sociodemographic factors influencing language development in young children.

## 1. Introduction

In 2020, approximately 98% of North American children aged 0 to 8 lived in households with various Internet-connected devices (e.g., television, tablets). Early exposure to technology may result in children spending, on average, more than two hours daily on screens [1]. As public health officials urged social isolation during the pandemic, more children became accustomed to screen-based life as families were increasingly reliant on technology for connection, learning, and entertainment. This increase in screen time has been linked to disrupted sleep patterns and poorer overall sleep quality in children [2].

Consequently, the American Academy of Pediatrics (AAP), the American Public Health Association (APHA), and the National Resource Center for Health and Safety in Child Care and Early Education (NRC) collaboratively developed a set of national guidelines known as Caring for Our Children (CFOC) based on research in child development, health, and safety [3]. Under Chapter 2 (Program Activities for Healthy Development) in Section 2.2.0.3, the CFOC guidelines stressed that children under 2 years should have no screen time, except for video chatting. Additionally, children 2 to 5 years of age should be limited to one hour of screen time daily, and screens should be removed from daily routines such as mealtimes and before bed, as well as during tantrums as a way of responding to children’s disruptive behaviors [3]. These guidelines are regularly updated as new scientific evidence emerges on the effects of screen exposure in early childhood [3].

The AAP also developed sleep guidelines based on a systematic review of research on child development, cognitive function, and health outcomes, in collaboration with the American Academy of Sleep Medicine [4]. Experts analyzed studies linking sleep duration to behavioral, cognitive, and physical health risks, using a consensus-based approach to determine optimal sleep ranges for different age groups [5,6]. As a result, the AAP recommended the following specific sleep durations: children 0 to 3 months should receive 14 to 17 h of sleep daily, children 4 to 11 months should receive 12 to 16 h, children 1 to 2 years old should receive 11 to 14 h, and children 3 to 5 years old should receive 10 to 13 h [7]. These guidelines emphasized that excessive screen time and inadequate sleep are negatively associated with a child’s development and may lead to behavioral and mental health concerns [8]. These recommendations are regularly updated to reflect new research on the impact of sleep on child health and well-being [7].

While there is a wealth of literature identifying the relationship between sleep and screen time on typically developing children and those with more severe delays, there is a gap in the research examining the correlation between sleep and screen time on language outcomes among young children with mild language delays. Language delays in young children affect up to 12% of children aged 2–5 years, and are associated with increased risk for future academic, social, and behavioral difficulties [9,10]. Risk factors include male sex, family history, low parental education, perinatal complications, and lower socioeconomic status [10,11]. Many children with language delays may catch up to their age group without interventions, but persistent delays (especially those involving both expressive and receptive language) are more likely to indicate a developmental language disorder (DLD) [12]. Early identification and intervention are crucial for these children, as persistent delays can have long-term impacts on language outcomes [13,14].

### 1.1. Sleep Duration and Language Development

Multiple longitudinal studies and meta-analyses indicate that longer and consistent nighttime sleep duration in early childhood is generally associated with better language development and expressive vocabulary scores at preschool [15,16,17,18,19]. Sleep consolidation (i.e., more night sleep relative to day sleep) also predicts better receptive vocabulary at school age [19]. Poor sleep consolidation or frequent awakenings are risk factors for language delay [15,16,18]. Napping plays a more nuanced role, as it is positively associated with vocabulary growth among infants and toddlers, but excessive napping at later ages may be associated with increased risk of language delay, especially in preterm infants [20,21]. Additionally, children with earlier bedtimes and consistent routines show higher expressive vocabulary [17].

While sleep supports overall development in all children, there is growing evidence that sleep difficulties may have a disproportionate impact on language development among children with or at risk of developmental delays. Specifically, children with developmental language disorders have been shown to experience more frequent sleep challenges, such as problems falling asleep and early waking, compared to their typically developing peers. In both children with developmental language disorders and their typically developing peers, difficulties initiating or maintaining sleep are strongly linked to poorer expressive, receptive, and social language outcomes [22]. Additionally, toddlers with developmental disabilities who experienced inadequate sleep also demonstrated greater deficits in language outcomes, particularly in vocabulary and syntax [23,24]. Children with typically developing patterns have been shown to have higher language abilities and better sleep behaviors, which may be further moderated by socioeconomic status and residence [22,25].

### 1.2. Screen Time and Language Development

Excessive screen exposure can lead to cognitive, language, social-emotional, and motor skill delays among children [26,27,28]. Research shows increased screen time is associated with poorer performance on developmental milestone assessments at 36 and 60 months of age among a diverse population of typically developing children [29]. Previous literature demonstrates that increased screen time (hours per day/week) and background television are linked to lower language skills, including delayed vocabulary acquisition and communication difficulties [29,30,31]. Additionally, studies suggest the timing of initial screen exposure plays a critical role in language development [26]. Earlier and excessive screen use (before 18 to 24 months) is particularly associated with poorer language outcomes [30,31,32]. Children introduced to electronic devices at 25–36 months of age experience fewer negative effects on language skills compared to those exposed at earlier ages [26].

Moreover, children with developmental delays spend more time viewing television compared to children without developmental delays [26,33,34]. The majority of children with language delays have been found to use digital devices (e.g., smartphones and tablets) [26]. In a study of Hispanic infants and toddlers aged 6 to 36 months, it was determined that of the participating cohort, those who watched more than 2 h of television daily tended to have lower communication scores [35]. Toddlers who used mobile devices for at least an hour a day tended to have poorer language development scores and receptive and expressive language delays [36].

Conversely, parent–child interaction is a critical factor that can buffer the negative impacts of screen exposure [37,38]. Children who interacted with their caregivers while watching television exhibited better expressive and receptive language abilities than children who watched alone [39]. Passive screen exposure, such as unsupervised television time, is more strongly associated with adverse language outcomes compared to educational programming and co-viewing with caregivers [38,40].

### 1.3. Relationship Between Sleep and Screen Time

Literature has demonstrated that sleep duration and both daytime and nighttime screen exposure are inversely related [2,41]. In a study of babies aged 3–6 months, it emerged that for every hour that an infant was exposed to nighttime electronic-based media, this resulted in the infant experiencing 13 min less sleep per night [41]. Similarly, toddlers had a delayed onset of sleep due to tablet exposure, and shorter nighttime sleep was indirectly associated with longer daytime naps [2]. Additionally, time spent watching entertainment content was significantly associated with poorer sleep quality and shorter sleep duration in children aged 2 to 5 [42]. These findings emphasize the importance of minimizing screen time and promoting healthy sleep habits to support children’s language development, particularly for those at risk of language and developmental delays.

### 1.4. Family and Sociodemographic Differences

Screen time and sleep duration in children are also associated with household and family factors, particularly education and income status. Education level typically intersects with socioeconomic status (SES), influencing access to resources such as stable housing and healthcare, which can mediate sleep quality [25]. One study revealed a positive correlation between children’s sleep duration, sleep quality, and the education level of their caregivers [43]. This finding sheds light on how higher household income was associated with better sleep quality and fewer issues with sleep [43]. Research following COVID-19 found that children in households with decreased income after the pandemic demonstrated a higher frequency and longer duration of use of smartphones and personal tablets [44].

Children in households where parents or primary caregivers have lower income and education levels have been shown to have shorter duration of sleep [45]. Furthermore, White children typically slept longer and had fewer naps compared to other races [46]. There is limited research on the relationship of other sociodemographic differences, such as ethnicity and insurance status, on screen time and sleep duration, particularly in urban settings. It is important to consider these variables to develop a more culturally sensitive approach to improving screen time and sleep duration in children.

While the relationship between family and sociodemographic factors and sleep and screen time has been examined in young children, little research has explored their associations specifically among children with mild language delays. It is crucial to understand the role family and sociodemographic factors play in screen and sleep time in children with mild language delays, as this may have implications for treatment.

### 1.5. Purpose of the Study

The purpose of this study was to examine the relationship between screen time and sleep duration on language outcomes in children with mild language delays. We utilized a novel approach to evaluate screen time and sleep by applying the most up-to-date nationally recommended guidelines from the CFOC for screen time and the AAP for sleep duration. Several studies have utilized similar approaches by incorporating screen time recommendations when analyzing their data (i.e., dividing their sample based on less than or greater than 2 h), but no studies to date have incorporated sleep guidelines [34,39,47]. Given the lack of recommendations specific to children with language delays, this study utilized widely accepted sleep and screen guidelines from the AAP for typically developing children to allow for a standardized benchmark for comparison. This study focused on children with mild language delays in an urban setting, given the limited research on this population and their atypical patterns of development. Although children with mild language delays may have unique developmental trajectories, they still require adequate sleep. Additionally, this study examined the role of various sociodemographic factors (i.e., ethnicity and insurance status) on the relationship between screen time and sleep duration.

The authors hypothesized as follows:

- Children with mild language delays who exceed recommended screen time guidelines will have lower expressive communication scores compared to those who meet the guidelines.
- Children with mild language delays who do not meet recommended sleep duration guidelines will have lower expressive and receptive communication scores compared to those who meet the guidelines.
- Sociodemographic factors, including lower household income, Medicaid insurance status, and lower caregiver education levels, will be associated with increased screen time and decreased sleep duration in children with mild language delays.
- Children with mild language delays who meet both AAP sleep guidelines and the CFOC screen time guidelines will have significantly better language development outcomes than those who do not meet one or both guidelines.

## 2. Materials and Methods

### 2.1. Early Discovery Program

Children who participated in the study were from the Early Discovery Program, a short-term intervention program for young children with mild developmental delays. The program was funded by The Children’s Trust, a non-profit organization that aims to improve the lives of children and families in Miami-Dade County. Local partners referred potential participants for early intervention. Families were assigned to one of four services based on screening results. Following intake, families were connected with the appropriate service provider. To examine the correlations between sleep and screen time on language development, only children with speech or language concerns were included in this study. Children completed the Preschool Language Scales, 5th Edition (PLS-5). Those who obtained a score of 71 to 84 (below average) in at least one domain were eligible to receive language intervention services. Interventionists tailored goals for treatment collaboratively with families based on findings from the PLS-5. Short-term services consisted of 8 to 20 sessions administered 1 to 3 times a week for 30 to 60 min over a 3-to-6-month period. Families were contacted monthly to discuss their children’s progress, ensure parent satisfaction, and integrate feedback accordingly.

### 2.2. Participants

This study included 765 children with mild language delays between the ages of 1 to 5 years and their families who enrolled in the Early Discovery Program from 2018 to 2021. Participants receiving language intervention services were administered a pre-test evaluation. Inclusion and exclusion criteria were established by the funding agency to meet the contractual requirements and deliverables specified in the service grant. Inclusion criteria consisted of having a child between the ages of 0–5, Miami-Dade County residence, and ineligibility for Part B or C services defined under the Individuals with Disabilities Education Act (IDEA). Additionally, children’s standard scores on the PLS-5 needed to fall within the below average range (i.e., scores between 71 and 84). The PLS-5 provides age-standardized scores, enabling reliable comparisons across children ages 1–7 years. Therefore, our analyses are interpreted based on each child’s performance compared to age-based norms. There was no indication of a regression in language acquisition; therefore, the challenges observed were interpreted as delays rather than deterioration [48,49]. Children with diagnosed disorders known to impact language development (e.g., hearing loss, ASD, cerebral palsy) were excluded from the study as they were eligible to receive Part B or C services. Children and their families who received program services other than language interventions were not included in this study.

### 2.3. Measures

Each family completed the Early Discovery Program Intake Packet at enrollment, a comprehensive tool created by program leadership. It included child and family demographics, such as race and ethnicity, language fluency, insurance coverage, and household income. Families also signed a participant agreement form prior to receiving services. A Strengths, Weaknesses, Opportunities, and Threats (SWOT) analysis was administered to identify family needs and strengths. While this instrument has not gone through formal psychometric validation, it has shown consistency in prior studies [50,51].

The following screen time and sleep duration questions from the SWOT were utilized in these analyses.

1. How many hours on average does this child sleep at night per day?
2. On a typical day, how much time is spent watching television, watching DVDs or videos, playing computer games, game consoles, or on an iPad or smartphone/tablet apps?

The analysis consisted of assessments evaluating developmental needs (i.e., delays), child health (i.e., sleep, screen time, etc.), and telehealth needs (i.e., access and use). Families also completed checklists on age-appropriate learning materials and home safety. Other questionnaires included the Family Quality of Life (FQOL) measure and Parenting Sense of Competence scales, which assessed overall family well-being, support, and caregiver-child interactions. Results were reviewed and utilized to guide interventionists in prioritizing need areas. Families were also connected to additional resources, as needed.

Children’s language skills were assessed at one timepoint and prior to intervention to reveal each child’s baseline level of performance using the PLS-5, a widely used measure designed for children up to 7 years old. The PLS-5 included two standardized scales, Auditory Comprehension (AC), and Expressive Communication (EC) as well as a Total Communication Score [52]. Children participated in a variety of structured tasks, which included naming objects, forming sentences, and various play activities. The PLS-5 has been shown to be particularly sensitive to mild language difficulties.

Child screen time and sleep duration were treated as binary variables (“Meets Guidelines” vs. “Does Not Meet Guidelines”), based on nationally recognized CFOC and AAP guidelines. Children met AAP sleep duration criteria and CFOC screen time criteria if they fell within the guidelines outlined in Table 1. This approach was chosen to facilitate interpretation and examine adherence in relation to established public health guidelines in children.

### 2.4. Statistical Analyses

Mixed approaches of univariate visualization and assessment of skewness and kurtosis were used to evaluate the normality of the numeric language outcome variables (PLS-5 Pre-AC Standard Score and PLS-5 Pre-EC Standard Score). Both skewness (between −2 and 2) and kurtosis (between −7 and 7) indices suggested that PLS-5 scores met acceptable thresholds for the normality assumption.

Descriptive statistics, including mean and standard deviation for continuous variables and count with percentage for categorical variables, were calculated for child and caregiver sociodemographic characteristics and PLS-5 scores (Table 2 and Table 3). Associations between screen time and sleep duration adherence and sociodemographic characteristics were examined using Pearson’s chi-squared test, Fisher’s exact test, or Wilcoxon rank sum test, as appropriate.

The impact of screen time and sleep duration guideline adherence on language development (PLS-5 scores) was examined using multivariable linear regression models, controlling for potential confounders including child gender, race, and ethnicity; caregiver gender, race, and ethnicity; caregiver highest education level; child insurance type; family household income; and number of siblings. Statistical significance was defined as a two-sided *p*-value less than 0.05.

To assess the robustness of findings and address potential residual confounding by age, a sensitivity analysis was conducted by additionally adjusting for child age (In months) in the multivariable models. These sensitivity results are presented alongside the primary model estimates in Table 3.

All analyses were performed using R statistical software version 4.3.2 [53].

## 3. Results

Results showed that among the 765 children included in the study, 383 (52%) did not meet the CFOC recommendations for screen time, and 487 (65.6%) did not meet the AAP guidelines for sleep duration (Table 2).

Children who did not meet AAP sleep guidelines were significantly more likely to identify as Black (child race: 21% vs. 9.9%, *p* = 0.003), have caregivers who identified as Black (caregiver race: 22% vs. 10%, *p* < 0.001), be insured through Medicaid HMO (child insurance status: 44% vs. 24%, *p* < 0.001), have a caregiver with a high school diploma or GED (caregiver highest education: 17% vs. 8.5%, *p* < 0.001), and reside in lower-income households (81% vs. 60%, *p* < 0.001).

Similarly, children who did not meet CFOC screen time guidelines were significantly younger (M = 32 months, SD = 27) than those who met the guidelines (M = 34 months, SD = 8; *p* < 0.001). They were also more likely to be non-Hispanic (child ethnicity: 20% vs. 14%, *p* = 0.049) and to have caregivers with a high school diploma or GED (highest education level: 17% vs. 12%, *p* = 0.03).

In unadjusted regression models, only child screen time adherence was significantly associated with EC scores. Children who did not meet CFOC screen time guidelines had significantly lower EC scores (β = −1.57, 95%CI [−2.66, −0.49], *p* = 0.004). This association remained significant and directionally consistent in multivariable models adjusting for sociodemographic variables (β = −1.73, 95%CI [−2.90, −0.57], *p* = 0.004). No significant associations were found between screen time and AC scores, either before or after adjustment. Sleep guideline adherence was not significantly associated with either EC or AC scores in unadjusted or adjusted models.

To assess whether age influenced the associations between screen time and sleep guidelines adherence and language outcomes, a sensitivity analysis was conducted by adding child age (In months) as a covariate to the multivariable models (Table 3). The association between non-adherence to screen time guidelines and lower EC scores remained statistically significant and directionally consistent (β = −1.75, 95%CI [−2.92, −0.59], *p* = 0.003). All other associations remained non-significant, with minimal changes in effect estimates, suggesting that child age did not meaningfully confound the observed relationships.

## 4. Discussion

This study utilized a novel approach by applying the most up-to-date nationally recommended guidelines, CFOC for screen time and AAP for sleep duration, to evaluate their associations with language outcomes in children with mild language delays. The authors also sought to understand the role of sociodemographic factors in these relationships. Findings demonstrated a significant portion of children did not meet sleep duration and screen time guidelines, which can exacerbate developmental concerns [54]. Screen time was negatively associated with expressive language abilities. Additionally, sociodemographic factors significantly influenced both screen time and sleep duration, highlighting their role in shaping developmental outcomes.

The CFOC recommends no screen time for those under 2 years, and no more than 1 h for those aged 2 to 5 [3]. Results indicated children with mild language delays exposed to excessive amounts of screen time were more likely to experience greater difficulties communicating with others compared to those who met guidelines. Contrary to previous findings [35,36,39], a significant relationship between screen time and receptive language scores was not found. This difference may be due to the quality of screen use, such as viewing educational content and co-watching with a parent, which may moderate the negative effects of screen time on language [39].

Additionally, the age at which children have their first exposure to screens may account for the absence of a significant relationship between screen time and receptive language scores. Findings evidenced that late-onset use of electronic devices (beginning at 25–36 months of age) is less detrimental to language development compared to early use. This suggests the timing of initial screen exposure can serve a vital role in moderating the effects of screen time on language skills [26].

This study and prior research emphasize the need for clinicians to evaluate and address screen time when working with families of young children with mild language delays, as excessive exposure has been shown to negatively impact brain structures involved in cognitive development [55]. Reducing screen time may be challenging for families, as caregivers may perceive it as educational or relaxing for their children [56]. Parent education programs designed to increase parental self-efficacy effectively decreased screen time in preschoolers [34,47]. Early intervention programs for children with mild language delays can encourage caregivers to limit screen time during language-directed activities such as meals and activities with friends and family.

According to AAP sleep guidelines, children should receive 10–17 h of sleep based on their age [7]. A significant association between sleep time and receptive or expressive communication scores was not found. A potential explanation is that our measure focused primarily on sleep duration, which may not encompass more nuanced aspects of sleep that impact language development, such as sleep quality or consistency of bedtime routines. It is also possible that sleep duration may not be as strongly connected to a child’s language development as screen time. Research suggests the quality of sleep could play a more significant role in language development than the number of total hours slept [22]. Additionally, the quality of language exposure may have a greater impact on a child’s development than sleep duration alone [57].

Given the high rate of noncompliance with sleep recommendations, it is essential for providers to work collaboratively with families when providing interventions, especially those targeting children with mild language delays, to ensure sleep is properly addressed. Establishing healthy sleep patterns may involve guiding families to make small, gradual changes to their screen time habits and bedtime routines. Engaging families in educational programs that offer practical strategies for improving sleep hygiene and managing screen time can encourage them to utilize more structured and consistent routines. Caregivers who set clear screen time limits and establish consistent bedtimes are more likely to have children who meet both sleep and screen time recommendations, supporting their overall development [58].

When considering the influence of sociodemographic factors, children’s race, household finances, and parental education were associated with both sleep and screen time. Consistent with previous studies, White children were more likely to adhere to recommended screen time and sleep guidelines compared to children of other racial backgrounds [45,46]. However, while race was significantly associated with sleep duration, no statistically significant relationship was observed between race and screen time. This discrepancy may be attributed to differences in the types of devices used (e.g., television, tablet, phone) as well as the quality of the screen time. Given this study did not assess the types of devices used by participants or examine the nature of interactions between the child and a parent during screen use, further research is needed to explore these potential contributing factors.

This study also highlights associations between SES, insurance status, screen time, and sleep. Children with mild language delays who resided in lower-income households (less than USD 70,000) were less likely to meet sleep guidelines compared to those in higher-income households (more than USD 70,000), suggesting a potential relationship between SES and sleep duration.

Additionally, significant differences in sleep duration were observed between children with Medicaid HMO and private insurance, with a greater number of Medicaid recipients not meeting current sleep guidelines. Interestingly, there was not a significant relationship between insurance status (i.e., none, Medicaid, CMS/KidCare, private) or household income (ranging from less than USD 70,000 to above USD 110,000) and screen time, as children across all groups were evenly distributed between meeting and not meeting guidelines. These findings emphasize the complex relationship between SES and health-related behaviors, particularly sleep.

Medicaid recipients tend to have a lower SES, which has been correlated with shorter sleep duration, potentially influenced by factors such as increased stress, unstable living conditions, and limited access to health resources [59]. The underlying mechanisms linking SES and child sleep duration remain unclear, though prior research suggests that SES-related differences may influence bedtime routines and sleep hygiene. Studies indicated caregivers from lower SES backgrounds may face challenges in establishing consistent bedtime routines, potentially contributing to later bedtimes for their children [60]. Conversely, caregivers from higher SES backgrounds may have greater access to resources that facilitate earlier bedtimes [61].

Insufficient sleep combined with socioeconomic difficulties may increase the risk of cognitive delays, academic difficulties, and behavioral concerns in children, potentially exacerbating family stress [61]. Furthermore, children from neighborhoods with limited resources tended to exhibit challenges across various aspects of language, including vocabulary and communication [25]. This may be mitigated by encouraging caregivers to engage in more frequent interactions and incorporate language-related activities, such as reading, from an early age.

Additionally, a statistically significant relationship was found between caregivers’ education level and both screen time and sleep duration, with higher education levels correlated with a greater percentage of children meeting guidelines. Prior research suggests that caregivers with higher levels of education reported shorter sleep latency in their children [62]. This may be due to greater awareness of health recommendations and increased access to resources that promote healthy habits [62]. Caregivers with a Bachelor’s degree or higher may be more likely to rely on expert guidance, textbooks, and pediatricians, whereas those with less formal education may turn to anecdotal evidence [62]. These differences in parental knowledge and access to reliable information also influence differences in child-directed language [62].

As clinicians work with families of children with mild language delays, it is essential to provide guidance that aligns with established recommendations while considering individual family needs. The CFOC and AAP guidelines should serve as a foundation for helping caregivers adjust their child’s sleep and screen time routines to better support development. Overall, the results emphasize the importance of early intervention programs that equip families with practical strategies to promote healthy sleep and screen time habits, ultimately improving children’s language outcomes.

### Implications

These findings suggest that screen exposure and sleep may influence language development potentially through environmental and cognitive pathways. The Bronfenbrenner and Morris (2006) bioecological model highlights how family context shapes routines and learning, while Vygotsky’s sociocultural theory (1978) underscores the importance of interactive communication [63,64]. Excessive screen use and inadequate sleep may disrupt attention and memory processes essential for language growth [65,66].

From a clinical perspective, these results underscore the importance of equipping early intervention providers with resources to counsel families on healthy screen and sleep habits. For example, providers can incorporate brief discussions on screen-free routines at mealtimes, bedtime, and playtime, and collaborate with caregivers to establish consistent routines. Incorporating these practices into early intervention programs may increase the child’s readiness for language learning. At a higher level, public programs for children with developmental delays could integrate modules on screen time and sleep into parent education. Pediatricians and early intervention programs can include screening questions about children’s media habits and sleep routines during well-child visits or intake assessments, allowing them to identify families who may benefit from targeted counseling. From a policy perspective, results support expanding early childhood programs to include caregiver training on healthy media use and sleep hygiene, particularly in communities facing socioeconomic barriers.

Finally, future studies should examine different aspects of sleep (i.e., quality and consistency) and screen time, as well as evaluate clinician interventions on overall language outcomes. It should also be noted that screen time was treated as one unity concept in the current study. Subsequent research should investigate the context and type of screen use [67,68], such as co-viewing or discussion of content, and the timing of screen exposure throughout the day. Longitudinal interventional research is also needed to determine whether modifying screen and sleep behaviors can meaningfully enhance language outcomes in early childhood. Additionally, future studies can analyze the impact of potential clustering effects, such as participants recruited from different neighborhoods.

## 5. Limitations

While this study provides important insights, several limitations should be considered. First, there was a potential for self-reporting bias as screen time may be underreported and sleep duration may be overreported by parents [69,70,71]. The SWOT analysis that measured screen duration and sleep time has not been formally validated; however, its previous use offered a standardized approach to data collection [50,51]. Although a formal a priori power analysis was not conducted, the relatively large sample of 765 participants likely provided sufficient power to detect meaningful associations. Nonetheless, future studies should include power calculations to further ensure the robustness of findings. It should also be noted that the current study cannot infer causal conclusions about the relationship between screen time and sleep, as the cross-sectional design limits the ability to determine directionality. Additionally, the sample was drawn exclusively from a county with a unique demographic composition, including a high percentage of Hispanic residents. However, it is considered broadly representative of the Miami-Dade population of children with mild language delays. The Early Discovery Program serves families throughout the county and reflects its demographic and linguistic diversity. Nevertheless, these characteristics do not reflect the overall US population, potentially limiting the generalizability of findings. The distinct cultural, socio-economic, and environmental factors in Miami-Dade County could influence results in ways that might not apply to other regions with different demographics. Future research should include a larger and more diverse sample from multiple regions to enhance the generalizability of findings.

## 6. Conclusions

This study highlights significant findings that support and challenge the current literature on key factors affecting sleep duration and screen time in young children with mild language delays. Findings revealed that most children with mild language delays are not meeting guidelines outlined for screen time and sleep, and that excessive screen time may negatively affect their expressive language skills. Additionally, race, insurance status, education level, and household income influence this relationship. Research should continue to examine the relationship between children’s sleep and screen time and their language, cognitive, emotional, and physical development to inform individual and effective, tailored interventions. It is recommended that clinicians adopt a multifaceted approach to guide families to utilize age-appropriate sleep duration and screen time recommendations to improve language outcomes for young children, especially among those with mild language delays.

## Figures and Tables

**Table 1:** AAP Sleep and CFOC Screen Time Guidelines by Age.

Age Group	AAP Sleep Duration Criteria (Hours/Day)	CFOC Screen Time Criteria (Hours/Day)
0 to 3 months	14 to 17	0
4 to 11 months	12 to 16	0
1 to 2 years	11 to 14	0
2 to 5 years	10 to 13	≤1

**Table 2:** Participants’ Demographics and PLS-5 Measurements by Child Sleep Duration and Child Screen Time.

Characteristic	Does Not Meet Sleep Guidelines, N = 487		Meets Sleep Guidelines, N = 255			Does Not Meet Screen Time Guidelines, N = 383		Meets Screen Time Guidelines, N = 382		
	n	%	n	%	*p*-Value ^a^	n	%	n	%	*p*-Value ^a^
Child Gender					0.400					0.600
Female	198	41	96	38		153	40	145	38	
Male	289	59	159	62		230	60	237	62	
Child Race					0.003 *					0.200
White	350	72	210	83		281	74	303	80	
Black	100	21	25	9.9		68	18	57	15	
Native American	0	0	0	0		0	0	0	0	
Asian/Pacific Islander	4	0.8	2	0.8		2	0.5	4	1.1	
Multiracial	25	5.2	11	4.4		23	6.1	12	3.2	
Other	5	1	4	1.6		5	1.3	4	1.1	
Child Ethnicity					0.400					0.049 *
Hispanic	348	71	187	75		273	72	286	75	
Non-Hispanic	89	18	38	15		75	20	52	14	
Haitian	32	6.6	12	4.8		22	5.8	21	5.5	
Other	16	3.3	13	5.2		10	2.6	19	5	
Unknown	2	0.4	0	0		0	0	2	0.5	
Parent Gender					0.600					0.600
Female	454	94	242	95		362	95	358	94	
Male	29	6	13	5.1		19	5	22	5.8	
Parent Race					<0.001 *					0.100
White	355	74	214	85		285	76	308	81	
Black	105	22	25	10		70	19	60	16	
Native American	0	0	0	0		0	0	0	0	
Asian/Pacific Islander	6	1.2	5	2		6	1.6	5	1.3	
Multiracial	9	1.9	6	2.4		11	2.9	3	0.8	
Other	6	1.2	1	0.4		5	1.3	2	0.5	
Parent Ethnicity					0.200					0.700
Hispanic	337	69	179	72		265	70	274	72	
Non-Hispanic	100	21	45	18		79	21	66	17	
Haitian	34	7	11	4.4		22	5.8	23	6.1	
Other	0	0	0	0		0	0	0	0	
Unknown	15	3.1	14	5.6		14	3.7	15	4	
Parent Education					<0.001 *					0.03 *
Elementary or less	3	0.7	0	0		2	0.6	0	0	
Some High School	20	4.4	5	2.1		13	3.7	13	3.6	
High School/GED	78	17	20	8.5		59	17	42	12	
Technical Training	20	4.4	4	1.7		13	3.7	12	3.3	
Some College	58	13	28	12		54	15	40	11	
Associates Degree	36	7.8	19	8.1		27	7.6	29	8	
Bachelor’s Degree	166	36	84	36		103	29	149	41	
Graduate Degree or higher	78	17	75	32		83	23	78	21	
Child Insurance					<0.001 *					0.140
None	8	1.7	3	1.2		5	1.3	8	2.1	
Medicaid (HMO)	211	44	62	24		136	36	149	39	
Medicaid (Medipass)	28	5.9	15	5.9		29	7.7	14	3.7	
CMS/KidCare	39	8.2	11	4.3		23	6.1	27	7.1	
Private	192	40	163	64		183	49	180	48	
Family Household Income					<0.001 *					>0.900
USD 0–70,000	382	81	147	60		278	75	276	74	
USD 70,001–110,000	30	6.4	39	16		34	9.2	34	9.1	
USD 110,001 and above	60	13	61	25		57	15	62	17	
	*M*	*SD*	*M*	*SD*		*M*	*SD*	*M*	*SD*	
Child Age (months)	33	24	32	10	>0.900	32	27	34	8	<0.001 *
Number of Siblings	2.04	1.11	1.81	0.86	0.035 *	1.95	1.06	1.95	1.00	0.700
PLS-5 Auditory Comprehension	89	14	90	14	0.500	89	14	90	13	0.300
PLS-5 Expressive Communication	79	8	79	8	0.300	78	8	80	7	0.004 *

Note: ^a^ Pearson’s Chi-squared test, Fisher’s exact test, Wilcoxon rank sum test. *: statistically significant.

**Table 3:** Impact of Child Sleep Duration and Screen Time on Speech Development Outcomes.

Outcome	Main Effect	Univariable Model			Multivariable Model ^a^			Sensitivity Model ^b^		
		Beta	95% CI	*p*-Value	Beta	95% CI	*p*-Value	Beta	95% CI	*p*-Value
PLS-5 Auditory Comprehension	Meets Sleep Guidelines	Ref.			Ref.			Ref.		
	Does Not Meet Sleep Guidelines	−0.37	−2.42, 1.69	0.73	0.89	−1.43, 3.13	0.47	1.02	−1.32, 3.24	0.41
	Meets Screen Time Guidelines	Ref.			Ref.			Ref.		
	Does Not Meet Screen Time Guidelines	−0.25	−2.17, 1.68	0.80	−0.43	−2.46, 1.67	0.71	−0.51	−2.52, 1.60	0.66
PLS-5 Expressive Communication	Meets Sleep Guidelines	Ref.			Ref.			Ref.		
	Does Not Meet Sleep Guidelines	0.45	−0.71, 1.62	0.44	0.84	−0.63, 1.96	0.32	0.87	−0.60, 2.00	0.29
	Meets Screen Time Guidelines	Ref.			Ref.			Ref.		
	Does Not Meet Screen Time Guidelines	−1.57	−2.66, −0.49	0.004 *	−1.80	−2.90, −0.57	0.004 *	−1.82	−2.92, −0.59	0.003 *

Note: ^a^ adjusting covariates of child gender, child race and ethnicity, parent gender, parent race and ethnicity, parent education, child insurance, and family household income. ^b^ additionally adjusted for child age (In months). *: statistically significant.

## Data Availability

The data presented in this study are openly available in Sleep and Screen Time Data (https://miami.box.com/s/rt3cpjrh029f2v7d0rua8kmt1rv582ku) and was accessed on 23 February 2022.

## Abbreviations

The following abbreviations are used in this manuscript:

AAP	American Academy of Pediatrics
CFOC	Caring for Our Children
SES	Socioeconomic status
PLS-5	Preschool Language Scales, 5th Edition
AC	Auditory Comprehension
EC	Expressive Communication
SWOT	Strengths, Weaknesses, Opportunities, and Threats
Ref	Reference
